# How to improve functional outcome of inflatable penile implant surgery? a narrative review

**DOI:** 10.1038/s41443-025-01030-9

**Published:** 2025-02-18

**Authors:** Ahmet Vural, Helene De Bruyn, Koenraad Van Renterghem

**Affiliations:** 1 Department of Urology, Cerrahpasa Faculty of Medicine, Istanbul University-Cerrahpasa, Istanbul, Turkey; 2 Department of Urology, University Hospitals Leuven, Leuven, Belgium; 3 Department of Urology, Hasselt University, Hasselt, Belgium; 4 Department of Urology, Jessa Hospital, Hasselt, Belgium

**Keywords:** Erectile dysfunction, Sexual dysfunction

## Abstract

The implantation of a three-piece inflatable penile prosthesis (IPP) has been shown to be a safe and successful treatment with a high satisfaction rate among individuals with erectile dysfunction. This narrative review aims to explore ways to improve the functional outcomes of IPP implantation. We conducted an English-language narrative review using all relevant articles sourced from PubMed. Over the years, modifications in IPP surgery have focused on increasing the longevity of prostheses and improving functional outcomes. These modifications include advancements in surgical methods, implant types, intracorporeal tubing length, the use of rear tip extenders, and reservoir placement. IPP implantation continues to significantly improve quality of life, making it essential for surgeons to stay updated on the latest developments and research to ensure the best outcomes for their patients. Optimal functional outcomes are achieved by an experienced surgical team and the use of a safe, rapid, minimally invasive surgical technique with the latest technology and equipment.

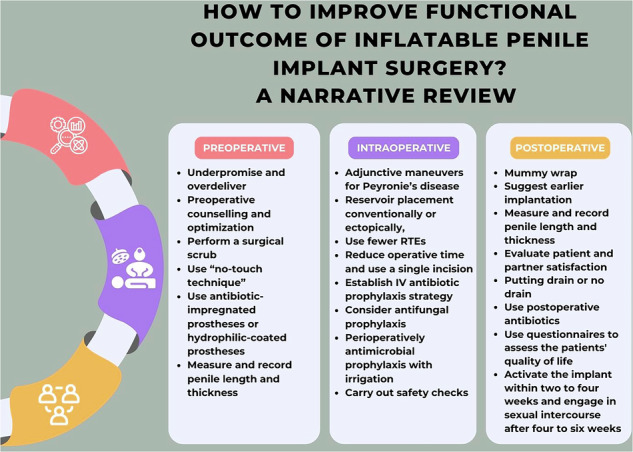

## Introduction

Individuals suffering from erectile dysfunction (ED) are unable to achieve and maintain an erection sufficient for satisfying sexual activity [1]. ED is a common disorder, affecting over 50% of males between the ages of 40 and 70 [2]. According to the European Association of Urology (EAU) guidelines on male sexual dysfunction, the implantation of a three-piece IPP is an effective and widely accepted therapeutic option for patients with ED, particularly when pharmacotherapy and vacuum devices are ineffective, unsatisfactory, or contraindicated due to comorbidities [3]. The 2020 EAU guidelines further recommend IPP implantation as an option when other treatments fail or based on patient preference [4].

Since the introduction of the first inflatable device by Scott in 1973, mechanical reliability has improved, and infection rates have decreased [5]. The most commonly used IPPs are manufactured by Boston Scientific (Boston Scientific Corporation, Marlborough, Massachusetts) and Coloplast (Coloplast A/S, Humlebæk, Denmark) [6]. Innovations in IPP cylinder design, materials, reservoirs, pumps, connections, and tubing have reduced mechanical failure and infection rates [7].

Enhancing surgical outcomes for IPP implantation can be achieved through innovations in device design and the refinement of surgical techniques [8]. Achieving optimal results in terms of patient safety and satisfaction requires a combination of both strategies. It is also crucial to recognize that the key to success lies in having a well-trained and well-organized surgical team [9]. A multidisciplinary team, including a urologist, cardiologist, endocrinologist, sexologist, and gynecologist, is also essential. Additionally, penile implant/prosthetic nurses and ward nurses provide indispensable paramedical support [10].

This narrative literature review aims to explore ways to improve the functional outcomes of IPP implantation, based on an extensive search of MEDLINE/PubMed to identify relevant articles.

## Literature search

A narrative review in English was conducted using all relevant articles sourced from PubMed in June 2024. The following keywords were used to guide the search: ‘penile prosthesis,’ ‘functional outcomes,’ ‘inflatable penile implant surgery,’ and ‘erectile dysfunction.’ Articles were selected based on specific criteria related to improving functional outcomes in IPP surgery. The most relevant articles were then included in the review. All non-English and non-full text articles were excluded. No publication date filter was applied. After the initial screening based on title and abstract, all papers were assessed in full text and excluded if deemed inappropriate (Table 1).

**Table 1:** Summary of the search strategy.

Items	Specification
Date of search	June 2024
Databases and other sources searched	PubMed
Search terms used	MeSH: penile prosthesis, functional outcomes, inflatable penile implant surgery, and erectile dysfunction
Timeframe	Due to a lack of evidence in some areas, all publication dates were considered, with a preference for articles from the last decade.
Inclusion and exclusion criteria	Inclusion criteria: clinical trial, meta-analysis, randomized controlled trials, reviews, and systematic reviews Exclusion criteria: Non-English language articles and articles with no full text
Selection process	All authors (A.V., H.D.B., and K.V.R.) conducted an extensive literature search independently. After merging all articles, duplicates were removed, and the most relevant articles were retained for the manuscript.

## Discussion

To improve IPP operations, we categorized our narrative review into preoperative, intraoperative, and postoperative measures. We also summarized strategies to improve the functional outcomes of IPP implantation in a table (Table 2).

**Table 2:** Summary of strategies to improve the functional outcomes of IPP implantation.

Preoperative	Intraoperative	Postoperative
- Underpromise and overdeliver - Show videos to patients demonstrating how the IPP works - Be clearly informed of the risks associated with IPP and ensure informed consent is obtained - Provide preoperative counselling and optimization - Select devices by evaluating clinical factors - Set postoperative expectations regarding penile length and sensation - Prepare the skin and perform a surgical scrub with alcoholic solutions such as isobetadine and chlorhexidine - “no-touch technique,” use sterile drapes to isolate the incision from the patient’s skin - Use antibiotic-impregnated prostheses (minocycline-rifampin) or hydrophilic-coated prostheses - Measure and record penile length and thickness	- Consider adjunctive maneuvers for patients with Peyronie’s disease - Place the reservoir conventionally or ectopically, if needed - Use fewer RTEs for better erection rigidity - Reduce operative time and use a single incision - Use the surgical approach with which you have the most experience for uncomplicated virgin patients - Provide for more proximalization by holding the urethra with Babcock tissue forceps - Establish the most appropriate IV antibiotic prophylaxis strategy (e.g., quinolone and co-amoxiclav) - Consider antifungal prophylaxis (e.g., fluconazole) - Irrigate the wound perioperatively with antimicrobial prophylaxis - Carry out safety checks	- Use the mummy wrap technique - Suggest earlier implantation to patients - Measure and record penile length and thickness - Evaluate patient and partner satisfaction following IPP implantation - Be informed that the use of a drain is still debated - Use postoperative antibiotics to prevent the development of infections - Use questionnaires to assess the patients’ postoperative quality of life - Activate the implant within two to four weeks and advise patients to engage in sexual intercourse after four to six weeks

*IPP* inflatable penile prosthesis, *RTE* rear tip extender.

### Incision type and preparation for surgery

Prevention of low satisfaction in patients and their partners begins with thorough and appropriate preoperative counselling. It is essential to assess the comorbidities of patients, discuss possible complications, and, most importantly, set realistic expectations [8]. Dissatisfaction often arises from inappropriate counselling and unrealistic expectations, particularly regarding postoperative sexual function. The most reported reason for dissatisfaction after penile implant surgery is the perceived loss of penile length [10]. Patients and their partners should be well-informed about the IPP, and the informed consent process should clearly outline the advantages and disadvantages of the surgery, alternative treatment options, costs, potential prosthetic complications, and expected clinical outcomes. Partners’ expectations should also be evaluated [11, 12]. Chronic conditions such as diabetes mellitus and cardiovascular comorbidities should be optimally managed before surgery to optimize surgical conditions [13]. Recent data on IPP implantation indicate that preoperative stretched penile length is a good predictor of postoperative penile length with no significant change in penile length postoperatively in either a deflated or inflated state. These findings are crucial for managing expectations preoperatively [14].

**Fig. 1:**
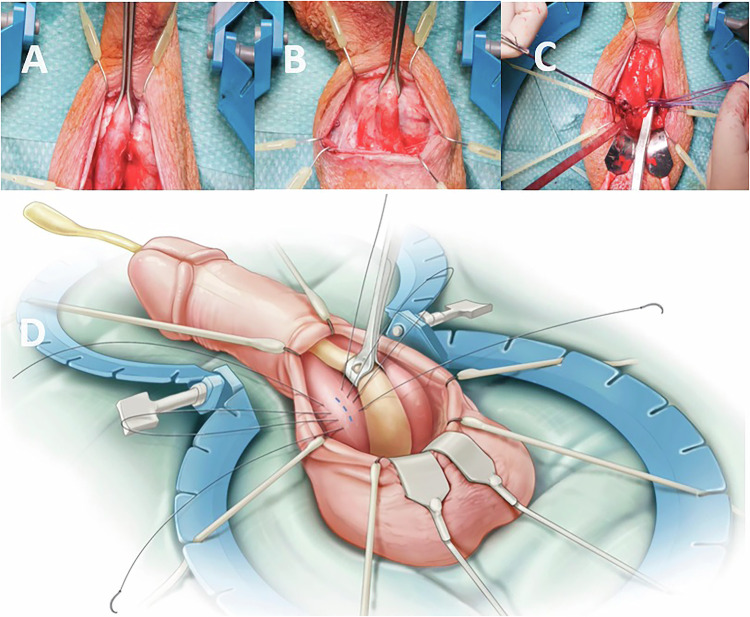
Penoscrotal Approach. **A** Using Babcock tissue forceps to hold the urethra. **B** Holding the urethra with Babcock tissue forceps to achieve greater proximalization. **C** Dilatation of the distal corpora cavernosa following corporotomy. **D** Placement of sutures in the corporal bodies and view of the corporotomy incision.

There are two main surgical approaches for penile prosthesis implantation: penoscrotal (PS) and infrapubic (IP) [15]. A single-center analysis of three-piece IPP implantation revealed no difference in satisfaction or complication rates between IP and PS placement [16]. The choice of surgical approach should be based on the patient’s anatomy, previous pelvic surgery, and the surgeon’s experience [17]. Both the IP and PS approaches are safe and have their own advantages and disadvantages [17]. Although experts continue to debate which technique yields the best outcomes, the PS approach is preferred by the majority of urologists for IPP surgery. Both approaches, however, have good patient satisfaction rates, with the IP incision remaining a safe and useful procedure [18]. The PS approach offers several advantages, including excellent exposure of proximal and distal corpora cavernosa, even in patients with obesity or corporal fibrosis, simplified pump placement, and a small scrotal incision resulting in a good cosmetic outcome [18]. Additionally, a double implant of an IPP and an artificial urinary sphincter can be performed safely with a single incision in patients with both ED and stress urinary incontinence refractory to conservative treatment [19]. In this method, if the urethra is damaged, the urethra can be easily seen and repaired during surgery. However, there is a risk of damage due to the blind reservoir placement (RP) into the space of Retzius (SOR) (Fig. 1) [9].

**Fig. 2:**
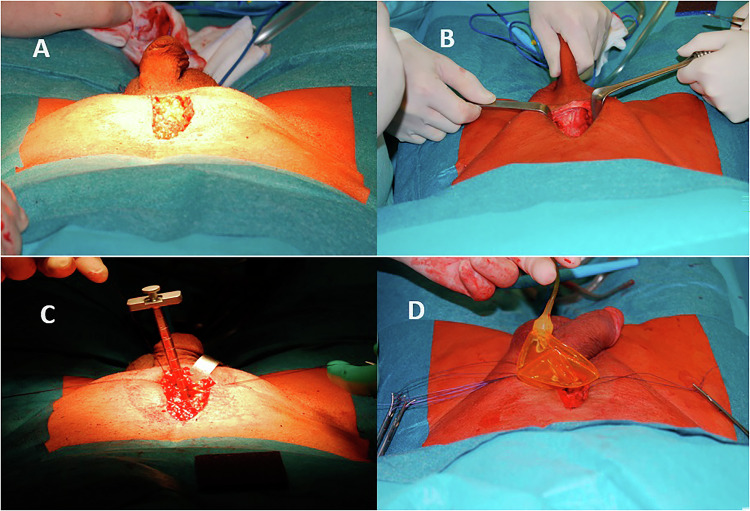
Infrapubic Approach. **A** View of the skin incision. **B** Exposure of the corporotomy site. **C** Measurement of the distal corporal length using the Furlow insertion tool. **D** Reservoir placement under direct vision.

The IP approach provides advantages such as easier and safer RP under direct vision with a small incision, reduced scrotal edema leading to faster pump activation, and a shorter operative time in experienced hands [20]. However, disadvantages include difficulty in pump placement due to possible pump migration, limited corporeal exposure, visibility of the IP incision scar, and an increased risk of injury to the sensory nerves of the penis. Additionally, revision surgery following the IP approach is associated with higher levels of difficulty and worse surgical outcomes (Fig. 2) [21]. Ultimately, the choice of incision type is largely the surgeon’s responsibility, based on their comfort, knowledge, and experience. The most critical goals for both the surgeon and patient to ensure a successful surgical outcome and a highly satisfied patient are setting realistic expectations and providing appropriate preoperative counselling, rather than the incision location [9, 15].

The subcoronal approach for IPP implantation allows for multiple reconstructive procedures to be performed safely and reliably with a single subcoronal incision, offering excellent visibility and access to the entire corporal body [22]. This approach remains a choice for IPP implantation in patients with severe Peyronie’s disease, including curvatures greater than 60°, severe indentation with hinge and grade 3 calcification, or those unlikely to respond adequately to manual modeling alone [23].

Both semi-rigid penile implants and IPPs each have their pros and cons. IPPs are generally preferred for their “natural” feeling and appearance, although there are no prospective randomized controlled trials comparing satisfaction rates between the two implant types [24]. The drawbacks of IPPs include mechanical complexity, which can lead to mechanical failure, and the need for good manual dexterity to use the device [25]. Additionally, the necessity of placing a reservoir in a three-piece system can be challenging for patients who have undergone previous pelvic surgery, often requiring alternative techniques such as the submuscular placement of the reservoir [26]. Semi-rigid prostheses are easier to use, particularly in patients with limited manual dexterity, but their main drawback is the continuous state of erection, which can be difficult to conceal [24]. Despite their complexity, IPPs are generally more attractive to the general ED population [27].

A study demonstrated that the use of IPPs led to shorter hospital stays and lower rates of perioperative urinary tract and surgical wound infections compared to semi-rigid penile prostheses [28]. Recent research also indicates that semi-rigid prostheses result in lower satisfaction rates than IPPs [29]. In the literature, three-piece IPPs have the highest satisfaction rates, with no significant difference in satisfaction between different brands of such prostheses [30]. Large prospective studies or registries, such as the PHOENIX trial, are needed to address unresolved questions in penile implant surgery. The PHOENIX trial, a multicentric survey on penile implant surgery conducted by the EAU Research Foundation, aims to collect data on routine surgical treatment procedures and short- and long-term follow-up outcomes of patients treated with the latest types of penile implant devices [31].

The use of a drain after IPP implantation remains a subject of debate [32]. However, a multicentric prospective non-randomized study conducted to investigate the risks and benefits of post-IPP implantation scrotal drainage and the optimal duration concluded that extended scrotal drainage for 72 h, compared to no drainage or 24-h drainage after virgin IPP implantation, significantly reduced hematoma and infection rates [33].

Single dilatation is a safer technique than multiple dilatations during IPP placement in uncomplicated virgin patients without fibrosis. It has been found to minimize postoperative complications, reduce corporal injury, and prolong the survival of the device without loss of cylinder length [34].

In a study in which Osmonov et al. explained what they learned from surgical body donor workshops, safety checks and landmarks that should be followed to reduce the likelihood of intraoperative problems during IPP implantation were outlined. The initial safety check is performed to rule out distal urethral perforation. Following distal dilatation, the corpus cavernosum is irrigated through the corporotomy. When the proximal dilatation is complete, both dilators are inserted into the proximal corpora, and the appropriate second safety check is conducted. Correct dilatation must be demonstrated using two dilators positioned at similar angles, plane, and depth. This could indicate that proximal corporal perforation or crossover dilation has been excluded [35].

### Mechanical complications and infection

Mechanical failure is a significant, though sometimes inevitable, complication of IPP implantation. Common causes of mechanical failure include auto-inflation, kinking of the tubing, cylinder aneurysm and rupture, fluid leakage, and pump dysfunction [36]. Some of these complications require the complete removal of the prosthesis, while others can be resolved with revision surgery. In a prospective multicentric analysis of 2384 patients, Wilson et al. reported the five- and 10-year overall mechanical survival rates for main implants to be 88.9% and 79.4%, respectively [37]. With technological advances, the risk of mechanical failure in IPPs has decreased over the years [37]. A large series, including 14,969 patients, concluded that the reoperation rate for non-infectious complications was 3.9% [38]. Patients should be informed that mechanical failure is inherently associated with a need for revision surgery, but the benefits of surgery should outweigh potential perioperative and postoperative complications [13].

Despite advancements in prosthetic devices and surgical techniques, infection remains a rare but serious complication of penile prosthesis implantation. High-grade infections can occur even years after implantation, although the infection rate for IPPs is typically low, reported to be 5% or less [39]. According to a study by Eid et al., the “no touch” surgical technique and infection-retardant coatings further decrease the rate of infection to 0.46% [40]. Risk factors for infection include diabetes mellitus, spinal cord injuries, immunosuppressed status (e.g., post-organ transplantation), and revision surgery [41]. Skin bacteria are the most common cause of infection, with the majority of isolated microorganisms being Staphylococcus species and Escherichia coli [42]. Improvements in surgical techniques, optimization of patient factors, and appropriate antibiotic prophylaxis, combined with the use of antibiotic or hydrophilic impregnated prostheses in high-volume centers, have significantly reduced the risk of prosthetic infection in low-risk patients [43].

There is heterogeneity among recommendations for perioperative antimicrobial prophylaxis in patients undergoing IPP surgery. The American Urological Association (AUA) recommends specific regimens, such as gentamicin plus vancomycin or a first- or second-generation cephalosporin [44]. However, the current AUA guidelines do not account for local resistance patterns and infection history and have poor anaerobic coverage [44]. The EAU previously recommended a second- or third-generation cephalosporin or a penicillin agent with anti-penicillinase activity [45]. Recent evidence, however, suggests that the regimens recommended by the AUA and EAU may be associated with higher postoperative infection rates than nonstandard regimens [46]. Consequently, the EAU no longer publishes specific guidance on perioperative prophylaxis, leaving surgeons to rely on the AUA’s recommendations [47]. The literature suggests that broadening antibiotic prophylaxis guidelines and establishing a management algorithm for IPP infections may reduce infection rates and increase the success of salvage outcomes [47].

In a multicenter study conducted by the Prosthetic Urology Multi-Institutional Partnership Collaborators in 2022, 4161 patients underwent primary IPP placement [48]. The study found that using vancomycin plus gentamicin alone for antibiotic prophylaxis was associated with a higher infection risk than non-standard antibiotic regimens, while adding an antifungal resulted in a lower infection risk. Including an antifungal such as fluconazole in the prophylactic regimen reduced the infection risk by 92%. These findings provide a strong rationale for the use of an antifungal alongside antibacterial agents in all patients undergoing IPP placement [48]. A critical review of antimicrobial prophylactic regimens based on local infection trends and antibiograms is needed, and prospective research should further elucidate best practices in IPP antimicrobial prophylaxis. The ongoing enrolled PHOENIX trial is anticipated to provide more insights into optimal antibiotic prophylaxis regimens for penile implant surgery [31].

Due to the severe consequences of IPP infections, surgeons follow strict infection prevention protocols, including shaving just before surgery, using antibiotic-impregnated prostheses (minocycline-rifampin) or hydrophilic-coated devices, preparing the area with surgical scrub (alcoholic solution, isobetadine, and chlorhexidine), employing no-touch techniques, perioperative wound irrigation with antimicrobial prophylaxis, administering high-dose antibiotics at induction (e.g., quinolone and amoxicillin), and minimizing operative time [49]. Although longer operative times are expected to increase the risk of IPP infection, the evidence is inadequate in the literature [41]. However, in a recent ex vivo study examining the effect of aerobiome exposure on IPPs in the operating room, the longer operative time did not increase the risk of bacterial growth on the IPPs [50]. Therefore, it is crucial for implant surgeons to manage operative duration carefully, which necessitates an experienced team and a dedicated nurse assisting in prosthetic surgery [15]. Furthermore, postoperative oral antibiotics may be prescribed at discharge by many surgeons to prevent possible IPP infections. When selecting postoperative oral antibiotic prophylaxis, factors such as surgeon preference, patient history, and local institution antibiotic recommendations are typically taken into account [51, 52]. However, there is no strong evidence from the literature that postoperative antibiotics can lower the incidence of IPP infection [51].

Localized penile prosthesis infections can be treated conservatively with antibiotics. However, if antibiotic therapy is ineffective, prosthesis removal is necessary to eradicate the infection. Delaying reimplantation may be advisable to promote proper wound healing and reduce the risk of reinfection, although it may significantly shorten the duration of erection and cause corporal fibrosis, resulting in the loss of penile length and further difficulties in subsequent prosthesis replacement [53]. In contrast, salvage penile prosthesis implantation involves the immediate reimplantation of a new prosthesis following the removal of the infected device and extensive wound irrigation with antiseptic or antibacterial solutions. Immediate salvage prosthetic surgery procedures have been shown to be safe and effective, allowing patients to resume sexual intercourse and preventing penile shortening [54]. In the literature, immediate salvage and removal of the infected device, followed by replacement with a new prosthesis, have been described using a protocol that achieved high success rates of over 80% for salvage procedures [55]. However, comparative studies evaluating the type of implant used during salvage procedures are needed to adapt conservative management strategies for optimal patient outcomes [56].

Another serious complication after penile prosthesis implantation is device erosion [57], which can occur independently but is often associated with infection [58]. IPP infection with systemic symptoms or in cases where erosion has caused the prosthesis to become visible requires device removal [59].

### Reservoir placement

The optimal RP in IPP surgery remains a debated issue. The gold standard procedure for males without prior abdominal surgery is the conventional RP [60]. An alternative procedure is the extraperitoneal retropubic SOR [26]. Serious complications associated with RP, such as bladder, vascular, or intestinal damage, reservoir herniation, and intravesical or intra-abdominal dislocation, have been reported, particularly in patients with prior pelvic or abdominal surgery. Therefore, there is an increasing research interest in the efficacy and safety of ‘ectopic’ or ‘alternative’ RP techniques, including intra-abdominal, high-submuscular, and even subcutaneous placement. Especially for patients with non-virgin pelvises, ectopic and mainly high-submuscular RP may be an ideal technique due to its safety, efficacy, and simplified learning curve [26]. While alternative or ectopic RP techniques are reliable even for patients with prior pelvic surgery, they carry risks such as reservoir leakage, tubing torsion, muscle irritation, and unexpected reservoir malposition, which may require surgical revision [61].

In a study conducted by Mykoniatis et al., 253 patients underwent IPP placement via a transverse PS incision using the SOR for RP. In the assessment of complications, only one patient reported prolonged pain (lasting one month) due to the reservoir. The SOR technique involves significant modifications that may affect the surgeon’s decision to use the classic RP. This approach, in which a fascial incision is made under direct visualization, also helps prevent complications such as blood vessel injury, inguinal hernia, and reservoir hernia [62].

### Corporotomy location and RTE use

The location of the corporotomy has direct implications for the basic and defining steps of IPP implantation, such as the surgical exposure of the corpora, corporal dilatation, IPP cylinder placement, RTE use, and tubing positioning [63]. Over time, friction caused by physical contact between components can lead to erosion and leakage of the cylinder, typically 12–18 months following implantation, a phenomenon known as ‘input tubing wear.’ Extended intracorporeal tubing can reduce the mechanical durability of the penile prosthesis and increase input-tube wear [64]. Performing a more proximal corporotomy incision could potentially resolve these issues, reducing the need for RTEs.

RTEs were developed in 1981 and quickly became popular among surgeons as a way to minimize tubing friction against the cylinder wall [65]. The reported high rate of RTE use, ranging from 58–73% during IPP surgeries, suggests that this innovation has provided surgeons with flexibility in corporotomy placement and minimized intracorporeal tubing [64]. However, some reports suggest that RTE use should be minimized, as it is associated with significantly higher revision rates and decreased penile rigidity during the inflation of the device. Additionally, increasing the length of RTEs has been shown to increase IPP bending deflection, which correlates with a decrease in axial rigidity [64, 66].

A laboratory study conducted by Thirumavalavan et al. on Coloplast prostheses found that longer RTEs were associated with greater bending deflection. Greater RTE length also limits the size of the IPP that can be implanted. Axial stress and bending deflection can affect the erect penis. This study supports the idea that maximizing inflatable length by minimizing RTEs improves overall erectile rigidity dynamics [67].

If the corporotomy’s proximal angle is near the tubing-cylinder junction, it might decrease the friction within the corporal body caused by the tubing pressing against the cylinder wall. The optimal corporotomy site varied depending on the type of prosthesis used. The ideal site is 3.3 cm proximal for the Boston Scientific AMS 700™ prosthesis (USA) and 4.4 cm proximal for the Coloplast Titan prosthesis (USA). The total cylinder length does not affect the proximal tip-to-tubing distance for either device. A corporotomy can be performed using various surgical techniques [68].

More proximal corporotomy incisions significantly reduce RTE use, but they are technically more challenging. The difficulty lies in reaching and appropriately dissecting this area, which can increase bleeding rates, as well as in dilating the corporal bodies distally and inserting the IPP cylinders with the Furlow tool. However, research has shown that, with experience, surgeons tend to perform the more proximal corporotomies [66].

### Patient and partner satisfaction

Patient satisfaction following IPP implantation is influenced by several factors, including patient expectations, comorbidities, partner attitudes, surgical complications, and premature device failures. Satisfaction is described as “an attitudinal response to the patients’ clinical encounter.” [69]. Furthermore, partner satisfaction plays a significant role, as it directly affects the patients’ sexual satisfaction. Various scoring systems are available to assess patient and partner satisfaction following IPP implantation. Commonly used larger questionnaires include the International Index of Erectile Function (IIEF) and the Erectile Dysfunction Inventory of Treatment Satisfaction (EDITS) [70, 71]. However, neither IIEF nor EDITS has been validated for use after IPP implantation. The only validated questionnaire for this purpose is the Quality of Life and Sexuality with Penile Prosthesis [72].

Very high patient and partner satisfaction rates for IPP implantation have been documented in the literature. Within six weeks after surgery, the majority of patients typically regain sexual function with minimal or no effect on orgasm. Jorissen et al. reported that more than 80% of patients were satisfied with their penile implant and would recommend the surgical procedure to a friend [27]. Recent research has shown that patient satisfaction remains high despite the development of mechanical failure and complications such as infection and erosion [73]. Preoperative counselling and shared decision-making help prevent lower overall satisfaction rates, minimizing the negative effect of postoperative dissatisfaction on functional outcomes. Over time, three-piece IPPs have consistently demonstrated high levels of satisfaction for both patients and their partners [4, 74].

Concerns about penis size are common among men considering IPP implantation, with many considering that their penis size may not be sufficient to satisfy their partner. The literature presents diverse views on this matter: while some women prefer a longer or wider penis, others find that penis size is completely unimportant during sexual activity. Although penis size can be a significant concern, it is not always the most important factor when assessing a partner’s level of sexual satisfaction [75].

Wilson et al. described penile modeling over an IPP as a crucial technique for patients with severe Peyronie’s disease. They found no evidence of penile shortening or impaired glandular sensation in patients who underwent this procedure [76]. However, men with severe Peyronie’s disease may require adjunctive maneuvers such as manual modeling, plication suture placement, or plaque incision (with or without grafting). In more severe cases of Peyronie’s disease, these procedures can lead to lower satisfaction with outcomes, particularly concerning penile length [77].

The most frequent complaint after penile prosthesis implantation is the loss of penile length, which can significantly affect overall patient satisfaction. To address this, it is important to discuss and establish realistic expectations during preoperative counselling [14]. According to research, earlier implantation of a penile prosthesis in patients with treatment-resistant ED has significant correlations with patient satisfaction, partner satisfaction, and whether the patient would recommend the procedure to a friend. Many patients and their partners have expressed regret for not having undergone IPP implantation surgery five years earlier [4].

## Conclusions

This review highlights the improvement of functional outcomes in IPP implantation surgery based on preoperative and postoperative findings. Despite the potential for various complications, the best functional outcomes are achieved by an experienced surgical team employing a safe, rapid, minimally invasive surgical technique with the latest technology and equipment.

## Data Availability

The data that support the findings of this study are available from the corresponding author (A.V.) upon reasonable request.
